# Tuberculosis Conveyed by Milk

**Published:** 1897-03

**Authors:** J. M. O’Neill

**Affiliations:** 852 Seneca Street


### Correspondence.

### TUBERCULOSIS CONVEYED BY MILK.

*To the Editor :*

Sir—I send the following account of some cases which have been brought to my notice, exemplifying the manner in which the bacilli of tuberculosis may be conveyed through the agency of milk. The details of the following cases have been supplied to me by a veterinary inspector, who was engaged on his duties in Cattaraugus county, some sixty miles distant from Buffalo. When there, he was requested by a farmer to inspect and test his two herds of cows. He complied with the request, and in the first herd, numbering eighty, he found eight to be infected with tuberculosis, and in the remaining herd he found twenty-five, out of a total of thirty animals, infected. A neighboring farmer then asked the inspector to test his herd. He did so, and found all healthy. The calves bred from some of these cows were then tested, and it was discovered that many were infected. The owner of the calves gave as a very plausible reason for their infection the fact that he was in the habit of buying skim- and buttermilk, with which to feed these calves, from farmers living in the immediate district, and among others from whom he procured this milk was the farmer whose herds the veterinary inspector had tested and found several of the cows to be suffering from tuberculosis. Of course, the foregoing account only goes further to prove the already well-known fact of the danger of spreading contagion by milk, and of the efficacy of the tuberculin test. In these particular cases, however, the danger affects Buffalo rather closely, for I also ascertained from the inspector that milk from these diseased herds was daily brought into Buffalo and sold on the streets by peddlers. The names of the peddlers was as a matter of course withheld from me. In connection with this statement I may say that I am at the present time attending a girl suffering from acute tubercular disease, and from whose family history and surroundings I have no doubt has been contracted probably by means of milk. Other instances of a like nature have occurred in my experience and I think it might be as well with regard to the safety of the inhabitants of Buffalo if stringent regulations were introduced in order to absolutely prevent the possibility of infected milk being sold in the streets, and thus remove any chance of the contagion being disseminated by such means.

J. M. O'NEILL, M. D.

852 Seneca Street.

